# On Food and Feeding

**Published:** 1904-09-24

**Authors:** Stuart Tidey


### Hospital Clinics.

### ON FOOD AND FEEDING.

By Stuart Tidey, M.D.

"Take no heed what ye shall eat nor what ye shall drink."

Regardless of the above authoritative injunction, no question exercises the mind of the average patient more imperiously than that of his dietary. The answers he receives are perplexing and contradictory with a general tendency towards the prescription of soft, highly nutritive, and pre-digested foods.

The most reliable advice occurs in the works of the immortal Elizabethan poet and scientist: "Now, good digestion wait on appetite, and health on both." This advice is very widely disregarded; the appetite is ruthlessly and systematically destroyed prior to birth, and remains an unfamiliar or unknown sensation.

The first rule, then, of dietetics is to encourage the appetite. Rigid dietaries, mush, slops and pre-digested foods find an occasional applicability in the treatment of disease, but their use approaches vanishing point as a consideration of the functions of the digestive tract overbalances the dogmata of dietetic orthodoxy, when weighed in the scale of experience.

What are the factors which tend to encourage an appetite?

In the first place, the digestive organs must be kept in a condition of cleanliness from end to end. In the second place, the various mechanical and physiological processes which subserve nutrition must be kept in a state of functional activity; for inactivity leads to physiological inertia and to structural decay.

Too often the stomach is treated as the dumping-ground for foreign products which should be manufactured at home, while the intestine is converted into a sedimentation tank, *quâ* excretion, when it should act as a periodically self-voiding sewer.

The teeth and mastication demand our first consideration. What lover of dogs having a puppy will allow him to grow up without bones to gnaw and hard biscuits to nibble. And yet, the same individual will demand for himself soft bread, tender meat, and every article of his dietary in such a state of pulp as to require no exercise of the tearing, cutting, or grinding functions of his dental armoury. And when his teeth commence to decay, he is surprised and asks the reason why.

The answer is "civilisation"—a term which expresses the angle of divergence between the path of Nature and that pursued by man under the dictates of custom.

What indication does Nature afford that we should expect our teeth to remain sound if we do not use them?

None! On the contrary, every unused organ undergoes retrogressive changes, becomes rudimentary and eventually disappears. Dental caries is the first step in the retrogressive process as it affects the teeth and jaws, and, should the cause remain active, we may anticipate the gradual disappearance of these organs and their substitution by a simple oral cavity into which predigested food may be poured.

Meanwhile, if we want to retain our teeth we must use them. Mastication of hard and solid substances tends to strengthen and develop the teeth and jaws; to harden, cleanse, and compress the gums; to stimulate the salivary and buccal secretions, thereby cleansing the mucous surfaces, and finally it gives the signal to the next important organ, the stomach, that a meal is on the road and to hold itself prepared to receive and deal with it.

Solid food, and hard solid food, should therefore form the bulk of human nutriment, and it should be well masticated. Personal attention to the teeth with the help of skilful dentistry go far to counteract the more serious symptoms of premature decay, but they do not, and cannot, supply the physiological stimulus which alone maintains an organ in a normal state of structural and functional perfection.

Renewed attention has been directed to the importance of prolonged chewing of food by Dr. van Someren. The principle is unquestionably right, and I find in practice that the artificial stimulation of the salivary and buccal glands by the use of chewing gum of great benefit where the mouth is dry, sticky, and foul.

Though not strictly belonging to the subject under consideration, yet in connection with the maintenance of buccal cleanliness, a word about the tonsils may not be inopportune. The frequency with which these organs become irritated and hypertrophied is probably associated with the general prevalence of insanitary conditions among civilised races. The tonsils are organs of elimination and possibly hypertrophy from excessive demands on their resources. The air we breathe is frequently foul and in cases where mouth-breathing is practised the foul air passes directly and continuously over the tonsils which must thereby be sorely tried. A foul mouth and carious teeth give rise both to chronic and acute tonsillitis and to my personal knowledge, the drilling of carious teeth will give rise to the same result. The tonsils, thus exposed to dangers arising from the unnatural conditions by which we are surrounded, need artificial aid to maintain their structural integrity and functional activity. By the habitual use of simple gargles, astringents and disinfectants, we can to a great extent counteract the dangers we cannot prevent.

To turn now to the stomach and its requirements. The decision as to how often it is desirable to take food is of the first importance. The modern tendency is to take four or five meals a day in health, and, when health has departed, to increase the number, so that the stomach is kept night and day in a state of functional fermentation.

On the other hand, Dr. van Someren flourishes on one meal in the 24 hours. Does the normal frequency lie within or without these limits for the majority of individuals? Is it more in accordance with our knowledge of physiology to approach a state of perpetual soaking in a nutritive medium, or to eat once in 24 hours and to allow the digestive organs long hours of repose between comparatively short periods of functional activity?

Arguing from analogy, it would seem that the muscular element of the intestinal tract would perform its function most effectually, if periods of activity were succeeded by periods of rest.

Assuming that the stomach occupies five hours in disposing of an average meal, during the first two hours of which it reaches its period of maximum activity whence it returns progressively to a state of rest, I should be inclined to allow it at least one additional hour of complete rest in order to restore it to a state of maximum digestive potentiality. This would give an interval of six hours between meals and admit of three meals a day and a long interval of repose during the night. If anything, this allowance errs on the side of excess. At the present time I have a patient who finds he can do hard manual work better on two meals a day than on three.

Apart from recuperation from muscular fatigue, the stomach and digestive organs generally may be assumed to require a period of functional inactivity in order to replenish the supply of digestive secretions. On such grounds I assume that three meals a day are amply sufficient to secure good nutrition, the stomach being allowed to rest in the intervals.

The question of what to eat derives importance from the prevalence of doctrinal dogmatism on the subject. The best rule is to eat everything, making exception only of such things as are obviously unwholesome or individually noxious. A general rule should be made of selecting hard food and masticating it thoroughly: stale bread, toast, tough meat, skin, pips, and the fibre of fruit and in general terms food which demands mastication and contains its nutritive elements in combination with fibrous, granular and non-nutritive matter.

The refuse matter plays the part of a wholesome excitant of muscular contractility, besides separating and distributing the nutritive portions, so that they may be dealt with by an extended area of mucous surface.

With such general hints and a recommendation to observe the promptings of idiosyncrasy, I leave the choice of dietary to the individual, insisting, however, on the desirability of eating plenty of vegetables and fruit.

I am inclined to think that in most individuals death is the result of eating to excess, and the end is usually hastened by misdirected attempts on the part of friends to bolster up the patient's strength by "feeding him up." The "feeding up" principle unfortunately meets with the support of the medical profession, which too frequently plays the part of a sounding board to the voice of popular prejudice.

But it is not the hypernutrition which characterises the last scene to which I wish now to allude, but the habitual consumption of an excessive quantity of food of a highly stimulating and nutritious quality, which not only overtaxes the digestive organs but throws a quantity of irritating matter into the blood. Thereby are set up organic changes which concentrate their effects on one organ or one set of organs, and lead to destruction of the whole through the defect of one part. Gout and rheumatism afford the most striking instances of what I understand by chronic nutritional poisoning. It is not the bulk of food consumed with which I find fault, but the practice of taking highly concentrated food, such as egg beaten up with milk and stiffened with brandy, strong beef-tea, meat extracts and the whole list of double-distilled death-dealers which adorns the columns of our daily press.

As a rule, therefore, in prescribing a dietary, I make a clean sweep of all concentrated articles of food including milk except in tea and coffee with non-nitrogenous meals. Next I insist on the selection of food in a solid form and of the hardest available.

Mastication follows as a necessity and prolonged mastication is recommended. Finally, the meals are limited to three a day with strict injunctions not to eat between meals.

Under such a system, the individual may select his dietary and eat his fill without any fear of gastric disturbance or plethora, and at the same time full nutrition will be secured and maintained.

With regard to drink, I prefer to exclude all forms of alcohol, allowing it however to adults who would miss it. In the case of adults, milk as a food is bad enough, in the guise of a drink I regard it as an abomination.

Water is the proper drink, taken as we give it our horses, before meals.

Half a tumblerful of water four times a day, taken on an empty stomach, quits that organ like a flash, enters the blood stream and flushes out the tissues generally and the kidneys in particular.

Some people seem to be unable to drink water unless they go to a spa, hence the importance of spas. The value of most mineral waters depends on the water as distinguished from the mineral. I once inquired into the mineral constituents of an Italian water, which from the time of the Cæsars had enjoyed a great reputation in the treatment of plethoric conditions, it proved to be as near an approach to pure water as any spring could conceivably furnish.

